# Bidirectional relationships between parenting stress and child behavior problems in multi-stressed, single-mother families: A cross-lagged panel model

**DOI:** 10.1111/famp.12796

**Published:** 2022-06-06

**Authors:** Qingyu Jiang, Dan Wang, Zhenqiao Yang, Jeong-Kyun Choi

**Affiliations:** 1 Department of Child, Youth and Family Studies, University of Nebraska-Lincoln, Lincoln, Nebraska, USA; 2 Department of Social Work, California Baptist University, Riverside, California, USA

**Keywords:** behavior problems, parenting stress, single mothers, transaction

## Abstract

Investigations on the bidirectional relationships between parenting stress and child behavior problems are important to inform intervention strategies; however, prior research has provided inconsistent findings. Using a national sample of multi-stressed single-mother families from the Fragile Families and Child Wellbeing study, the present study examined the bidirectional relationships between maternal parenting stress and children’s behavioral problems spanning from early childhood through adolescence at the child’s ages 3, 5, 9, and 15. Reciprocal transactions were found between parenting stress and behavior problems in early childhood between the ages 3 and 5. From age 5 to age 15, our findings also suggest that children’s behavior problems at an earlier time point predict mothers’ parenting stress at a later time point. Unexpectedly, the lagged effects of parenting stress on child behavior problems in school ages were not significant in our sampled data. Early childhood interventions should address mitigating both parenting stress and their toddlers’ behavior problems. During middle childhood and adolescence, interventions to directly address children’s behavior problems are critical both to the well-being of mothers and to assist in the reduction in levels of behavior problems.

## INTRODUCTION

Parenting stress is a negative psychological reaction to the demands of the parenting role (Deater-Deckard, 1998), which results from a mismatch between perceived parenting demands and available parenting resources to meet these demands (Abidin, 1990). Parenting stress has been found to have a detrimental effect on children’s behavioral development (Carapito et al., 2018; Mackler et al., 2015), whereas increased behavior problems raise challenges that can contribute to increased parenting stress (Beernink et al., 2012; Woodman, 2014). Prior research has shown the significant associations between parenting stress and behavior problems across developmental periods, including early childhood (Anthony et al., 2005), middle childhood (Neece & Baker, 2008), and adolescence (Anderson, 2008). These unidirectional investigations, however, do not provide a valid causal inference. Furthermore, inconsistent results have been found in longitudinal studies, which investigated the bidirectional relationships between parenting stress and child behavior problems (Cherry et al., 2019; Neece et al., 2012; Rodriguez et al., 2019; Woodman et al., 2015). Questions still remain about the causal relationship between parenting stress and child behavior problems, particularly in socioeconomically disadvantaged families.

The present study examined how parenting stress and child behavior problems longitudinally influence one another from early childhood to adolescence, using a subsample of multi-stressed single-mother families from the Fragile Families and Child Wellbeing study (FFCWS). Previous research has mostly focused on two-parent married families. Socioeconomically disadvantaged families are often underrepresented in research samples. Parenting stress in single motherhood is well documented (Berryhill & Durtschi, 2017). The challenges associated with personal, social, and financial resources in a single-mother household can be a stressor for both the mother and her children. Children living in multi-stressed families are at greater risk of having poor discipline (Damaske et al., 2017; Escudero & Friedlander, 2017) and problematic behaviors (Harmeyer et al., 2016; Wang et al., 2020a; Wang et al., 2020b). Nonetheless, few studies have focused on understanding the stability and trend in parenting stress of single mothers with limited resources over time. The transactional associations between parenting stress and children’s behavior problems in multi-stressed single-mother families have been understudied. The practical value and utility of furthering our understanding of potential bidirectional associations between parenting stress and child outcomes are expected to offer the justification for informed decisions to set age-specific goals and priorities for future intervention. Extending such knowledge potentially may inform practice and policy efforts to support socioeconomically disadvantaged families and their children. Therefore, we sought to extend current knowledge by (a) using longitudinal panel data collected at the child’s ages 3, 5, 9, and 15 with a sample of single mothers and their children that experienced economic hardships and (b) investigating the bidirectional relationships between parenting stress and children’s overall behavior problems as well as internalizing and externalizing behaviors spanning from early childhood through adolescence.

## Parenting stress and child behavior problems

There is substantial evidence that parenting stress is associated with children’s behavior problems in the literature that has posited unidirectional associations (Anthony et al., 2005; Crnic et al., 2005; Deater-Deckard, 1998). Parenting stress is specifically associated with poor parenting behavior (Pelchat et al., 2003), less responsive and more controlled practices (Ward & Lee, 2020), and more coercive and harsh parenting (Choi & Becher, 2019). These stress-led parenting practices provide a negative impact on children’s behavioral development (Deater-Deckard & Scarr, 1996). Particularly for low-income single mothers, their parenting stress is directly related to more frequent behavior problems of their focal child; however, this association is often mediated by less competent and more abusive parenting (Jackson & Huang, 2000; Jackson & Choi, 2018; Jackson et al., 2019). Another body of research suggests a reversal of this relationship, showing that children’s behavior problems are linked to elevated parenting stress across time (Beernink et al., 2012; Hastings, 2002; Tervo, 2012). The results of hierarchical linear models indicate that child behavior problems predict initial status and increase in parenting stress in early childhood (Williford et al., 2007). In a longitudinal dyadic study spanning from early childhood through adolescence, children’s behavior problems are associated with higher levels of parenting stress perceived by both mothers and fathers; however, children’s adaptive behavior was not significantly associated with mothers’ parenting stress (Woodman, 2014). Consistent with these findings, a study focusing on single mothers with limited resources reveals that their children’s negative emotionality predicts parenting stress trajectories over time in early childhood (Berryhill & Durtschi, 2017).

Bidirectional associations between parenting stress and child behavior problems have been examined; however, there are mixed findings. With a sample of 237 families—including 93 children with developmental delays, Neece et al. (2012) investigated bidirectional relationships between parenting stress and child behavior problems, annually assessed across seven time points from ages 3 to 9. Four out of the six cross-lagged effects from parenting stress and behavior problems were significant. Early parenting stress was observed to predict later behavior problems between the ages 3 and 5, as well as between the ages 6 and 7. Early child behavior problems were shown to predict later mothers’ parenting stress between the ages 5 and 6, along with between the ages 8 and 9. Cross-lagged effects were significant between the ages 7 and 8 (Neece et al., 2012). In a similar vein, a study from Woodman et al. (2015) recruited a sample of 176 families of children with developmental disabilities to examine transactional relations between mothers’ parenting stress and child behavior problems over 15 years from the child’s age 3 to age 18. Significant cross-lagged effects were observed for parenting stress and internalizing behavior in early childhood (ages 3–5) and adolescence (ages 10–15). Internalizing behavior was found to predict parenting stress in middle childhood (ages 5–10). Externalizing behavior was also observed to predict parenting stress from ages 5 to 15, whereas the pathway from parenting stress to externalizing behavior was found significant from ages 15 to 18 only (Woodman et al., 2015). Using data of three annual waves from mothers of 1582 children aged 4 to 7 at wave 1, Stone et al. (2016) examined transactional relations between parenting stress and children’s internalizing and externalizing problems. Significant cross-lagged effects were observed for parenting stress and externalizing problems from ages 4 to 9. Mothers’ parenting stress predicted internalizing problems between the waves 1 and 2 only (Stone et al., 2016). Additionally, Rodriguez et al. (2019) examined bidirectional effects between parenting stress and behavior problems, annually assessed across four time points within 188 families of children aged 5 to 12 with autism spectrum disorder. All three cross-lagged effects from mothers’ parenting stress at an earlier time point to later internalizing problems were significant; however, internalizing problems were not found to predict later parenting stress at any time point. On the contrary, early parenting stress was observed to predict later externalizing problems for the first three waves, whereas the cross-lagged effects from externalizing problems to parenting stress between the waves 3 and 4 were significant (Rodriguez et al., 2019). The sampled children did not belong to the same age cohort group; therefore, the results should be interpreted with caution.

In summary of these aforementioned studies, transactional relations have been found between parenting stress and children’s overall behavior problems as well as internalizing and externalizing behaviors in early childhood. The transactional model by Sameroff and Chandler (1975) supports that both child and parent factors have reciprocal influences on one another since early childhood. This suggests that bidirectional associations between parenting stress and child behavior problems occur in the early years of a child’s life. Regarding child-driven effects, internalizing behaviors predict parenting stress in early childhood to middle childhood, and externalizing behaviors predict parenting stress in middle childhood to adolescence. These patterns of findings are aligned with a process model of the determinants of parenting (Belsky, 1984), suggesting the influence of children may be predictive of parental functioning (e.g., parenting, parenting stress, and parents’ psychological well-being) throughout development. A recent meta-analysis by Yan et al. (2021) has also revealed that children’s externalizing behaviors are positively linked to parental stress and intrusive and harsh parenting. In terms of parent-driven effects, parenting stress predicts both internalizing and externalizing behaviors in middle childhood and adolescence.

These aforementioned studies with a focus on children with developmental concerns have yielded inconsistent findings. The data came from predominantly married households in the middle- or upper-income categories. The sampled participants were mostly white, highly educated, and from middle or high socioeconomic status (SES) groups. Recruiting a sample of low-SES families and children at risk for behavior problems, Mackler et al. (2015) examined the longitudinal transactions between parenting stress and child externalizing behavior problems at the ages 4, 5, 7, and 10. The results confirm the transactional model, indicating that cross-lagged effects between parenting stress and externalizing behavior were significant in all time points. Regarding effect sizes, the effects of early externalizing behavior on later parenting stress were overall greater than those of early parenting stress on later behavior problems. The data were collected from a single university-based laboratory, and the study participants were largely white; thus, the findings are not representative. Recently, Kochanova et al. (2022) examined reciprocal relationships between parenting stress and children’s externalizing and internalizing behaviors among 1209 low-income families with their children in early childhood (aged 2–5) or in early adolescence (aged 9–15) at time 1, tested three time points over 6 years. In the early childhood cohort, parenting stress predicted child internalizing behaviors between time 2 and time 3. In the early adolescence cohort, child internalizing behaviors predicted parenting stress across three time points. However, bidirectional associations were observed between parenting stress and child externalizing behaviors in the early adolescence cohort only (Kochanova et al., 2022). Cherry et al. (2019) also tested transactional models for parenting stress and child behavior problems, using a sample of low-income families and their children enrolled in Early Head Start programs. Hispanic or African American mothers (69%) and welfare recipients (72%) were a substantial part of the participants. Bidirectional associations between parenting stress and behavior problems were significant between the ages 1 and 2; however, no unidirectional or bidirectional associations were found between the ages 2 and 3. Attrition was one of the study’s limitations, as were the findings, which were limited to young children under the age 3.

Despite rigorous investigations, the literature is inconsistent regardless of unidirectional or bidirectional associations. Furthermore, bidirectional associations between parenting stress and behavior problems on vulnerable populations—that are given high priority to policy and intervention—are still understudied. Even a few studies on socioeconomically disadvantaged families have limitations such as the use of unrepresentative samples or the limited coverage of developmental periods. To fill this gap, the present study used a nationally representative sample of multi-stressed single-mother families to investigate bidirectional associations between mothers’ parenting stress and their children’s overall behavior problems as well as internalizing and externalizing behavior across developmental stages spanning from early childhood to adolescence.

## Conceptual framework

Parenting stress, according to Hill’s (1958) family stress theory, occurs when the demands associated with parenting outweigh the parent’s abilities and resources (Abidin, 1983; Cherry et al., 2019; Parkes & Sweeting, 2018). A child’s behavior problems (e.g., tantrum, aggression, defiance) can be an additional demand. When compared to parents of children with fewer behavior problems, parents of children with more problematic behaviors experience higher levels of stress (Arikan et al., 2019; Barroso et al., 2018; Cherry et al., 2019; Lee et al., 2011; Solem et al., 2011). Additional stressors (e.g., economic hardship) or inadequate support (e.g., the absence of father involvement) may prevent parents from coping with their child’s demands and problems, causing their parenting stress to become a crisis. According to the family stress model (Conger et al., 2000), multiple stressors (e.g., economic hardship, single parenthood) may contribute to parents’ psychological distress and parenting stress, which can, in turn, disrupt children’s positive development. This model views a child’s behavior problems as an outcome of parenting stress, rather than a source of stress for parents. Evidence shows that unmarried mothers often experience poverty, economic hardships, and a lack of support (Damaske et al., 2017; Jocson & McLoyd, 2015). These stressors may elevate mothers’ parenting stress, and children raised by mothers with high levels of parenting stress are more likely to demonstrate disruptive and problematic behaviors (Wang et al., 2020a; Wang et al., 2020b). The transactional theory of human development (Sameroff & Chandler, 1975; Sameroff & Fiese, 2000) posits that children develop within interactions primarily between children and their parents where they simultaneously influence each other, leading to a transactional relationship. As a stressor, behavior problems may increase parental stress and negatively affect parenting practice, which, in turn, increase the risk of developing problematic behaviors. Reciprocal transactions may occur between parenting stress and child behavior problems over time (Cicchetti & Schneider-Rosen, 1984; Morgan et al., 2002; Sameroff & Mackenzie, 2003).

**FIGURE 1:**
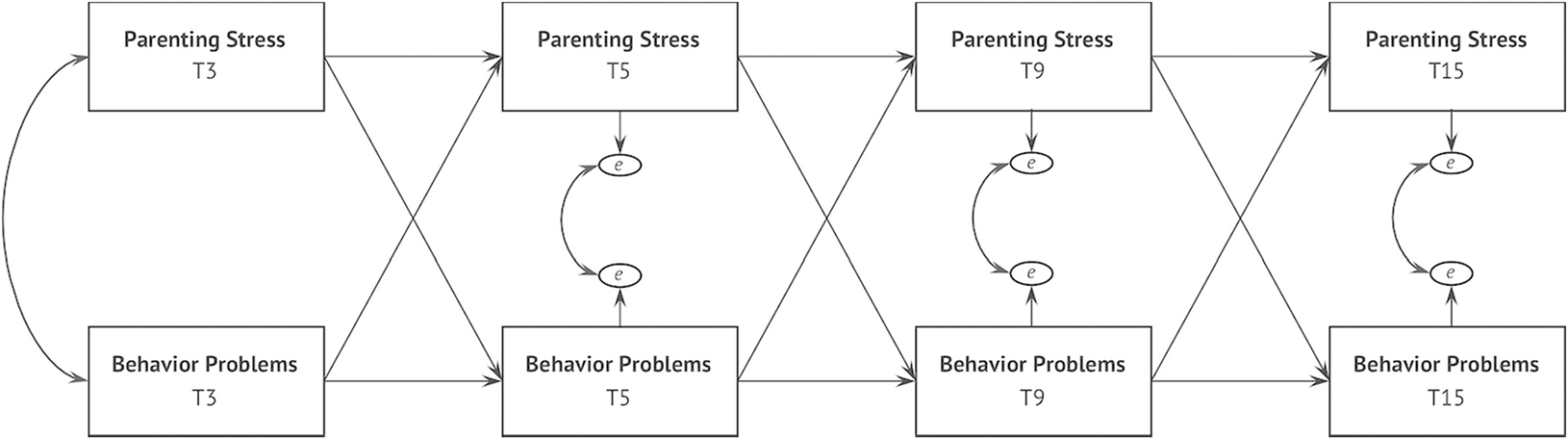
Conceptual model

The present study aims to test these theoretical perspectives. Figure 1 depicts the conceptual model that we propose. Informed by these perspectives, we hypothesized that (a) more children’s problematic behaviors at earlier times would result in higher levels of mothers’ parenting stress at later times; (b) higher levels of mothers’ parenting stress at earlier times would lead to more behavioral problems in children at later times; and (c) bidirectional associations would occur between parenting stress and child behavior problems over time. With a focus on socioeconomically disadvantaged families, we used a national sample of multi-stressed single-mother families from the FFCWS to investigate the bidirectional relationships between maternal parenting stress and children’s overall behavioral problems as well as internalizing and externalizing behavior spanning from early childhood through adolescence, assessed at multiple time points at the child’s ages 3, 5, 9, and 15.

## METHODS

### Data and sample

We used longitudinal data from the FFCWS that had over-sampled children born to unmarried parents and followed a birth cohort of 4898 children (The Trustees of Princeton University, 2021). A considerable number of Black, Hispanic, and low-income families were included in the sample. The baseline and follow-up surveys, interviews, and home observations were conducted at the child’s birth and ages 3, 5, 9, and 15. Among the initial sample, we selected 888 children from unmarried mothers who had experienced poverty. Mothers who have married or never been poor for the first 15 years of a child’s life were excluded. There were 118 mothers who had never reported their parenting stress and children’s behavior problems; therefore, the final sample of this study consisted of 770 mothers and their children. The demographic and socioeconomic characteristics of the sampled mothers and their children at the baseline (wave 1) are shown in Table 1. More than half of the sampled mothers (61%) were non-Hispanic Black, followed by Hispanic (20%) and non-Hispanic White (16%). Mothers were on average 23.8 years old (SD = 5.6). Teen mothers accounted for 25%; those between the ages 20 and 24 were 41%; and those between the ages 25 and 29 were 20%. Over 40% of mothers (41%) had some high school education; around a third had a high school diploma or an equivalency degree; and less than a quarter (23%) had college education. More than half of mothers (54%) received public support or welfare benefits. Mothers’ annual income was on average $8260 (standard deviation = 7558.5). There were marginally more boys (52%) than girls in the sampled children.

### Measures

#### Maternal parenting stress

Fragile Families and Child Wellbeing study measured mothers’ parenting stress using four items adapted from the Parent Stress Inventory (Abidin, 1995) and the primary caregiver of target child household questionnaire in the Child Development Supplement of the U.S. Panel Study of Income Dynamics (Hofferth et al. 1999). In the follow-up surveys at the child’s ages 3, 5, 9, and 15, mothers were asked to rate the degree to which they agreed or disagreed with the following statements: (a) being a parenting was harder than she had thought, (b) she felt trapped by her responsibilities as a parent, (c) taking care of her child was much more work than pleasure, and (d) she often felt tired, worn out, or exhausted from raising a family. The response options were 0 (strongly disagree), 1 (disagree), 2 (agree), and 3 (strongly agree). Item scores were averaged to create composite scores of maternal parenting stress at each time point. The internal consistency reliability for this scale was marginally acceptable, indicating that Cronbach’s alphas were 0.59 for age 3, 0.64 for age 5, 0.64 for age 9, and 0.62 for age 15. Using only a few items in this scale, we also reported the average interitem correlation (AIC). In the current sample, the AIC was 0.37 for age 3, 0.43 for age 5, 0.43 for age 9, and 0.41 for age 15. All of these imply that the items have homogeneity and also distinctive variance so as not to be redundant (Clark & Watson, 2019; Piedmont, 2014).

#### Child behavioral problems

The preschool and school-age versions of the Child Behavior Checklist (CBCL) were used in FFCWS to assess the focal children’s behavioral problems at the child’s ages 3, 5, 9, and 15. Mothers were asked to indicate the frequency or intensity of behavioral problems with the following statements about whether the focal child: (a) acted too young, (b) destroyed things, (c) did not get along with other children, (e) got in many fights, (f) cried frequently, (g) had angry moods, (h) showed little affection toward people, (i) had speech problems, (j) could not concentrate, and (k) wanted excessive attention. A 3-point Likert scale was used with the response options ranging from 0 (not true), 1 (sometimes or somewhat true), to 2 (very true or often true). Item scores were averaged to create composite scores of child behavioral problems at each time point. FFCWS included 65 items for ages 3 and 5 from the preschool version of the CBCL/1.5–5 (Achenbach & Rescorla, 2000), as well as 124 items for age 9 and 32 items for age 15 from the school-age version of the CBCL/6–18 (Achenbach & Rescorla, 2001). The internal consistency reliability for this scale was good or excellent, indicating that Cronbach’s alphas were 0.92 for age 3, 0.88 for age 5, 0.95 for age 9, and 0.91 for age 15. Higher scores indicate more frequent or intensive behavior problems.

#### Covariates

We tested the following covariates at the baseline (wave 1): child gender and mothers’ age, marital status, cohabiting status, education level, and race/ethnicity; however, the models showed either no convergence or poor model fits. These covariates were not included in the final models.

### Analysis

Descriptive and correlational analyses were conducted using STATA 14.2/SE (StataCorp, 2015). Autoregressive cross-lagged coefficients were estimated using structural equation modeling with Mplus 7.4 (Muthén & Muthén, 1998–2017). The variables included in our final models contained 13.9% missing data, on average, ranging from 11.2% to 35.9%. Results from the Little’s (1988) missing completely at random test indicated that the data were not missing at random (χ^2^ = 292.3, degrees of freedom = 246, *p* = 0.023). Full information maximum likelihood was used to address missing data, given that it is a less biased and efficient practice than ad hoc missing data methods (Newman, 2014). As shown in Figure 1, autoregressive paths were estimated for both parenting stress and children’s behavioral problems across the four time points at the ages 3, 5, 9, and 15. Cross-lagged pathways and transactional effects were estimated between parenting stress and children’s behavioral problems over time.

## RESULTS

Table 2 presents a matrix summary of the correlations, means, standard deviations, skewness, and kurtosis. Due to non-normality, parenting stress and behavior problems were converted to corresponding square roots and percentiles, respectively (Hobbs & King, 2018; Lee & Jackson, 2017; Wang et al., 2020a, Wang et al., 2020b). Mean scores show that parenting stress decreased over time whereas behavior problems increased from 3 to 5 years of age and subsequently decreased from 5 to 15 years of age. All bivariate correlations were positive and significant (coefficient *r* ranged from 0.151 to 0.592). The presence of multicollinearity was not detected.

**FIGURE 2:**
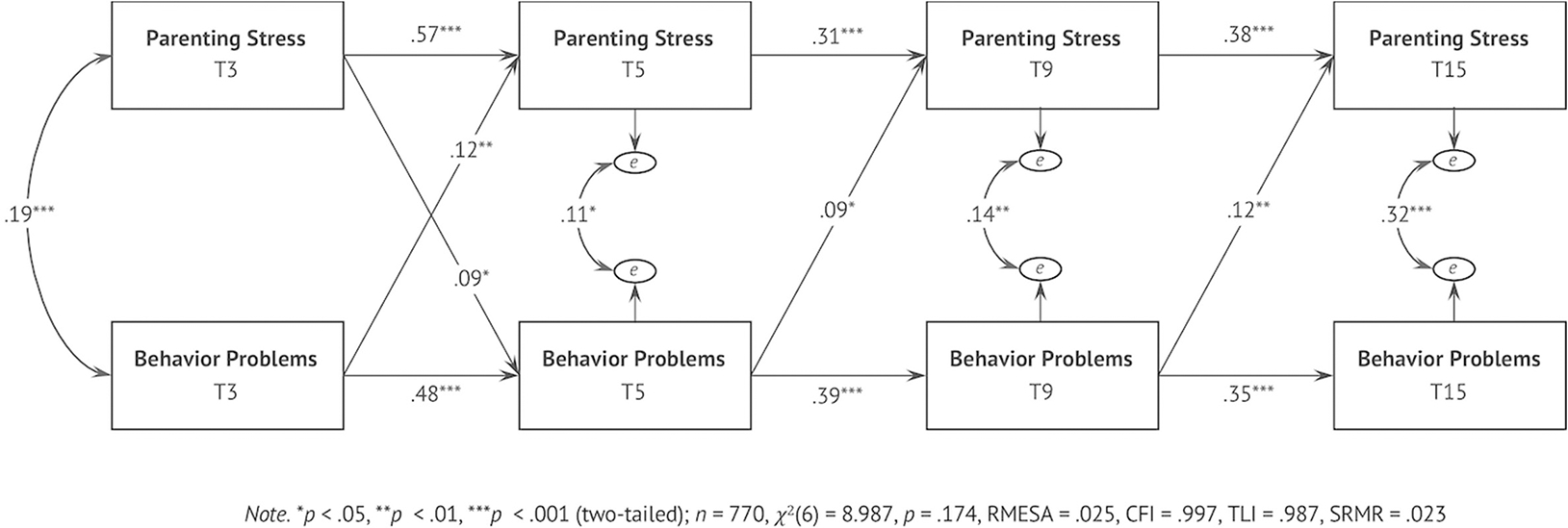
Final structural equation model (*n* = 770). All hypothesized paths (in Figure 1) were analyzed. Non-significant paths and their coefficients were not displayed in this figure

The final cross-lagged panel model in Figure 2 shows a chi-square of 8.987 with 6 degrees of freedom (*p* = 0.174), a root mean square error of approximation of 0.089, a comparative fit index of 0.997, a Tucker–Lewis index of 0.987, and a standardized root mean square residual of 0.023. All model statistics indicate a good fit to the data. Figure 2 also shows the standardized coefficients for bidirectional relationships between parenting stress and behavior problems. Significant transactional effects were observed in both directions between the ages 3 and 5. In other words, parenting stress at age 3 predicted behavior problems at age 5 (*β* = 0.09, *p* < 0.05) and behavior problems at age 3 also predicted parenting stress at age 5 (*β* = 0.12, *p* < 0.01). Regarding effect size, the effect of behavior problems on parenting stress was larger than that of parenting stress on behavior problems. No significant transactional effects between the ages 5 and 15 were observed. Behavior problems at age 5 predicted parenting stress at age 9 (*β* = 0.09, *p* < 0.05), whereas the reverse path from parenting stress at age 5 to behavior problems at age 9 was not significant. From age 9 to age 15, early behavior problems predicted later parenting stress (*β* = 0.12, *p* < 0.01); however, the path from early parenting stress to later behavior problems was not significant. Among the hypothesized cross-lagged paths, all three paths from behavior problem at an earlier time point to parenting stress at a later time point spanning from early childhood to adolescence were significant. In contrast, the only significant pathway from early parenting stress to later behavior problem was observed in early childhood from age 3 to age 5. Autoregressive paths for parenting stress show a high degree of stability, indicating that all three pathways from parenting stress at an earlier time point to that at a later time point were significant (*β* = 0.57 from age 3 to age 5; *β* = 0.31 from age 5 to age 9; *β* = 0.38 from age 9 to age 15). Autoregressive paths for behavioral problems were also significant (*β* = 0.48 from age 3 to age 5; *β* = 0.39 from age 5 to age 9; *β* = 0.35 from age 9 to age 15). The correlation between parenting stress and behavioral problems both at age 3 was significant (*r* = 0.19, *p* < 0.001). The correlations among error terms of parenting stress and behavioral problems at age 5 (*r* = 0.11, *p* < 0.05), at age 9 (*r* = 0.14, *p* < 0.01), and at age 15 (*r* = 0.32, *p* < 0.001) were also significant.

### Sensitivity analyses

**FIGURE 3:**
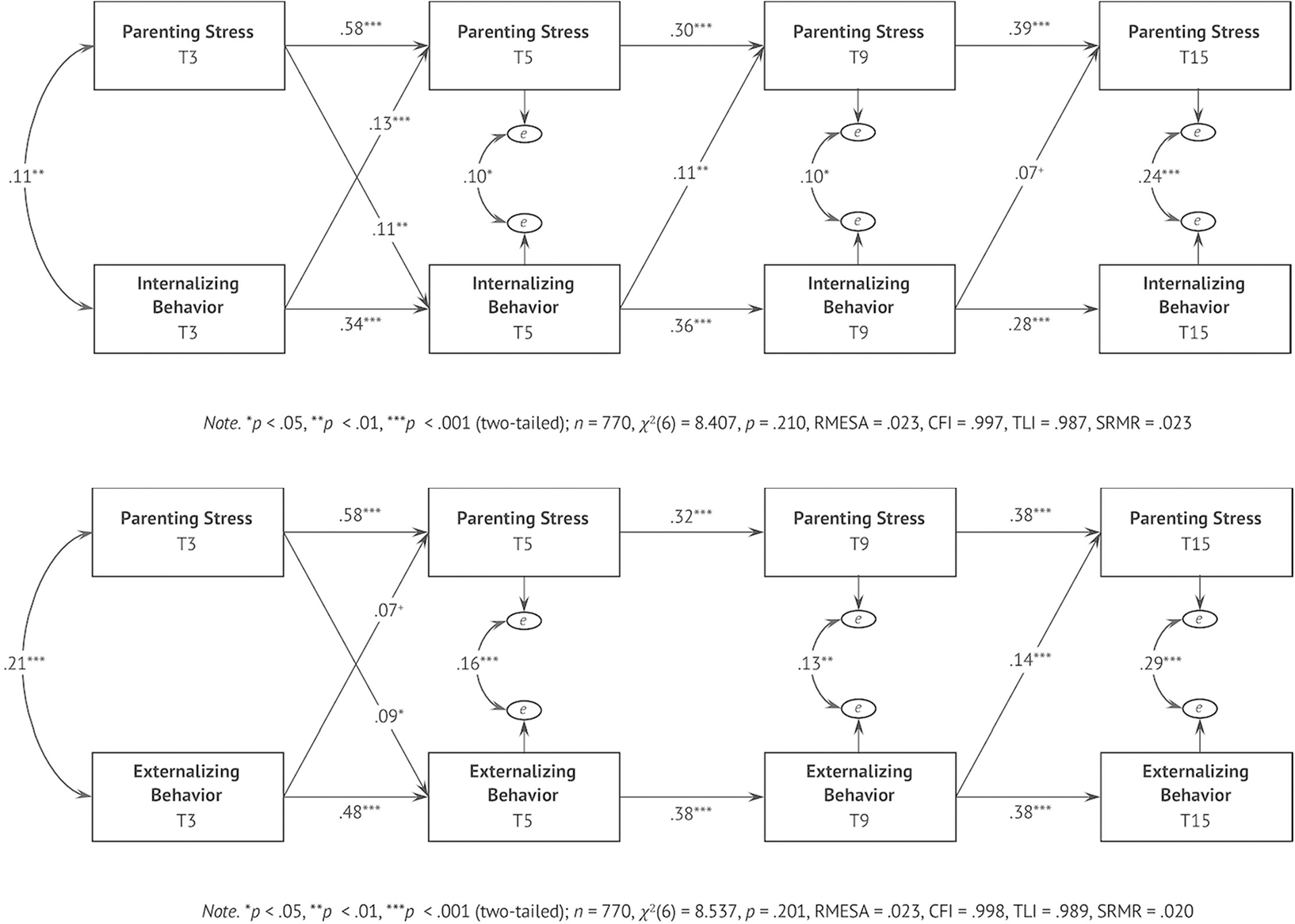
Internalizing and externalizing behavior models (*n* = 770). All hypothesized paths (in Figure 1) were analyzed. Non-significant paths and their coefficients were not displayed in this figure

Sensitivity analyses were conducted to evaluate alternate models. We separated internalizing and externalizing behavior from the behavior problems scale. Both internalizing and externalizing behavior at the same period of time from age 3 to age 15 were measured to test alternate models and determine differences in findings. As shown in Figure 3, both internalizing and externalizing behavior models fit the data. All pathways in these alternate models remained similar to the original model. In both models, cross-lagged effects between parenting stress and each of internalizing and externalizing behavior were significant from age 3 to age 5. Internalizing behavior at an earlier time predicted later parenting stress from age 5 to age 15. Externalizing behavior at age 9 also predicted parenting stress at age 15. However, the pathway from externalizing behavior at age 5 to parenting stress at age 9 was not significant, which differed from the original model.

## DISCUSSION

The current study examined the bidirectional relations between parenting stress and children’s behavioral problems across the child’s ages 3, 5, 9, and 15 in multi-stressed, single-mother families. Reciprocal transactions were found between parenting stress and behavior problems in early childhood from age 3 to age 5. This is consistent with transactional theory (Sameroff & Chandler, 1975; Sameroff & Fiese, 2000), as well as support for the transactional effects between parenting stress and internalizing behavior in early childhood (Woodman et al., 2015; Mackler et al., 2015). This finding also shares similarities with previous studies indicating bidirectional relations are observed between parenting stress and child overall behavior problems as well as between parenting stress and internalizing behavior in the preschool years (Neece et al., 2012; Woodman et al., 2015). This finding of the present study extends the understanding of simultaneous influences between mothers and children who are experiencing financial strain and single parenthood.

From age 5 to age 15, our findings suggest that children’s behavior problems at an earlier time point predict mothers’ parenting stress at a later time point. This result is also aligned with prior literature demonstrating the cross-lagged effects of behavior problems on parenting stress for school-aged children (Neece et al., 2012) and adolescents (Woodman et al., 2015). It should be noted that a child’s problematic behaviors are a critical stressor for mothers (Arikan et al., 2019; Barroso et al., 2018; Cherry et al., 2019; Lee et al., 2011; Solem et al., 2011), particularly when they have already suffered from poverty, economic hardships, and the lack of support. Furthermore, a positive association from child internalizing behavior at age 5 to mothers’ parenting stress at age 9 was found. This finding is consistent with Woodman et al.’s (2015) study in which child internalizing behavior predicts parenting stress from age 5 to age 10. In addition, our results indicated that children’s externalizing behavior at age 9 predicted mothers’ parenting stress at age 15. This pattern matches the finding of Kochanova et al.’s (2022) study in which child externalizing behavior predicts parenting stress in middle childhood to early adolescence.

Unexpectedly, the lagged effects of parenting stress on child behavior problems over school ages were not significant in our sampled data. This result supports that mothers’ parenting stress does not escalate their children’s behavior problems in childhood and adolescence, which is incongruent with the family stress model (Conger et al., 2000). In other words, the current findings indicate that a child’s behavior problems are not a consequence of mothers’ parenting stress but a contribution to their stress in socioeconomically disadvantaged families. To summarize, the present study supports the conclusion that children’s behavior problems are predictive of consequent parenting stress across developmental periods from early childhood to adolescence. Early childhood was the only time point when reciprocal relations between parenting stress and behavior problems were observed in the current data. This may be explained by the transactional model assuming parents and children bidirectionally and mutually influence each other in early childhood (Sameroff & Chandler, 1975). That is, child behavior problems may rise parental stress and are negatively related to parenting practice, reciprocally, elevated parenting stress may result in behavior problems in early childhood (Morgan et al., 2002; Sameroff & Mackenzie, 2003). Unidirectional associations from behavior problems to parenting stress were found in middle childhood and adolescence in our study. It is possible that older children display more behavior problems and externalizing behavior compared with young children (e.g., defiance, disruption, fights, and attacks). As a result, these challenging behaviors may increase parents’ stress (Allen et al., 2010; Yan et al., 2021). Likewise, internalizing behavior in adolescents may generate greater stress for parents due to the higher likelihood of the longitudinal influences of mental health difficulties (Kochanova et al., 2022).

Although the present study advances current understanding of the relationships between parenting stress and child behavior problems, the findings should be interpreted within the context of its limitations. Mothers’ strengths or resiliencies that could be coping mechanisms considering their at-risk status were not included in our conceptual model. While focusing on the home environment, we did not consider school or neighborhood contexts and rule out these external influences. In relation to measures, only four items available in the FFCWS dataset were used to assess mothers’ parenting stress and its internal consistency reliability was marginally acceptable. Although confirmatory factor analysis showed that all four items were acceptable indicators with loadings higher than 0.4, a comprehensive scale to measure multi-dimensions of parenting stress should be considered. Moreover, demographic covariates were not included in our final models due to poor model fits. Future studies may include other covariates in addition to demographic covariates. Furthermore, all variables used in this study, including child behavior problems, were based on mothers’ self-reported perceptions only. Utilizing only mother-reported data would introduce some common method variance. Research on informant effects suggests that adding additional informants increases the validity of statistical results when investigating relations between parental perception and children’s internalizing and externalizing problems (Izquierdo-Sotorrío et al., 2016). Future research may consider incorporating direct observations or measures from other caregivers’ reports such as grandparents, fathers, or teachers. Regarding data analysis, it should be acknowledged that cross-lagged models predict individual differences but do not account for within-individual changes over time. The within-person effect was not examined in the study. For example, deviations in parenting stress from the parent’s typical levels may also be associated with deviations in child behavioral problems from a child’s typical levels. Future research may examine both between-person and within-person effects using alternative models, such as the random-intercept cross-lagged panel model (Orth et al., 2021). Additionally, it should be noted that the length of the time between waves is an important issue in longitudinal research. This might result in non-significant cross-lagged effects in our findings. Key constructs in the FFCWS were measured at the child’s ages 3, 5, 9, and 15; therefore, the length of the interval between two consecutive waves ranges from 2, 4, and 6 years. Short interval cross-lagged effects would be more prominent and detectable than those in long interval ones. We understand that FFCWS chose these particular time points of observation based on children’s developmental stages (e.g., newborn, infant, toddler, preschool, school-age, and adolescence). Future researchers should take into account this issue throughout the study design and analytic phases (Taris & Kompier, 2014).

Despite these limitations, our findings have implications for interventions to support multi-stressed, single-mother families and their children. Interventions adapted to specific development phases can be effective. Given the reciprocal influences between mothers and children in early ages, interventions should address mitigating both parenting stress and their toddlers’ behavior problems (Woodman et al., 2015). These aims have been incorporated into evidence-based programs to improve parent–child interaction such as the Parent–Child Interaction Therapy (Zisser & Eyberg, 2010) and the Triple P-Positive Parenting Program (Turner & Sanders, 2006). Another invention goal in early childhood should be empowering mothers to cope with stress and respond to challenging behaviors. Strengthening mothers’ coping skills—through psychoeducation on conflict resolution, optimistic parental value structures, and positive self-esteem (Parent et al., 2016), as well as mindfulness practices and relaxation strategies (Lunsky et al., 2017)—can be an effective approach to alleviate the negative impact of parenting stress on their parenting behaviors and potential conflicts with children. During middle childhood and adolescence, interventions to directly address children’s behavior problems are critical both to the well-being of mothers and to assist in the reduction in levels of behavior problems in adolescents themselves. For instance, the Positive Action program is an evidence-based practice that aims to promote self-concept, positive action, emotional management skills, responsibility, and social interaction. The Promoting Alternative Thinking Strategies curriculum is another effective strategy for improving self-awareness, self-management, social awareness, relationship management, and responsible decision-making. These school-based interventions for elementary and middle school students have shown to be successful in reducing children’s misconduct and improving their social–emotional skills (Domitrovich et al., 2017; Duncan et al., 2017).

In conclusion, investigations on the bidirectional relationships between parenting stress and child behavior problems are important to inform intervention strategies. The present study discovered that mother–child transactions were simultaneous only in the early childhood period. During middle childhood and adolescence, the effects of behavior problems on parenting stress overweighed those of parenting stress on behavior problems. Our findings serve as an initial step in understanding the reciprocal relations between parenting stress and child behavior problems in multi-stressed, single-mother families. More empirical studies are still needed to further investigate these complex transactional relations in broader environmental contexts.

## Figures and Tables

**TABLE 1:** Mothers’ demographic and socioeconomic characteristics (*n* = 770)

Variables	Frequency	Percent

Mother’s race/Ethnicity		
White	124	16.2
Black	468	61.2
Hispanic	153	20.0
Asian and other	20	2.6
Mother’s age		
15–19	190	24.7
20–24	315	40.9
25–29	152	19.7
30–39	99	12.9
40–43	14	1.8
(Mean, SD)	(*M* = 23.8)	(SD = 5.6)
Mother’s education		
Some high school or less	314	40.9
High school diploma or GED	262	34.1
Some college or 2-year degree	175	22.8
Bachelor’s degree or higher	17	2.2
Work hour		
Unemployed	48	6.2
19 h or less	110	14.2
20–29 h	123	16.0
30–39 h	429	55.7
40 h or more	60	7.8
(Mean, SD)	(*M* = 34.7)	(SD = 10.4)
Welfare receipt		
Recipient	415	54.0
Child’s gender		
Boy	398	51.7
Annual income		
$4999 or less	278	46.1
$5000–9999	137	22.7
$10,000–19,999	126	20.9
$20,000 or higher	62	10.3
(Mean, SD)	(*M* = $8260.4)	(SD = 7558.5)

**TABLE 2:** Correlation, mean, and Standard deviation of variables (*n* = 770)

Variable	1	2	3	4	5	6	7	8

1. Parenting stress (T3)								
2. Parenting stress (T5)	0.592***							
3. Parenting stress (T9)	0.416***	0.459***						
4. Parenting stress (T15)	0.334***	0.424***	0.454***					
5. Behavior problems (T3)	0.207***	0.248***	0.150**	0.158***				
6. Behavior problems (T5)	0.200***	0.281***	0.193***	0.216***	0.502***			
7. Behavior problems (T9)	0.102**	0.173***	0.215***	0.219***	0.396***	0.482***		
8. Behavior problems (T15)	0.128**	0.164***	0.151***	0.388***	0.219***	0.383***	4.11***	
*N*	684	625	593	601	568	493	553	601
Mean	1.39	1.35	1.22	1.19	6.19	6.25	6.16	6.06
SD	0.64	0.68	0.70	0.68	2.72	2.79	2.78	2.92
Skewness	0.24	0.06	0.28	0.31	−0.27	−0.27	−0.28	−0.24
Kurtosis	2.77	2.63	2.61	2.69	1.95	1.84	1.91	1.78

* *p* < 0.05

** *p* < 0.01

*** *p* < 0.001.

## References

[R1] Abidin RR (1983). Parenting stress index: Manual, administration booklet, [and] research update. Pediatric Psychology Press.

[R2] Abidin RR (1990). Parenting stress index-short form. Pediatric Psychology Press. 10.1093/jpepsy/10.2.169 4020601

[R3] Abidin RR (1995). Parent stress inventory (3rd ed.). Psychological Assessment Resources.

[R4] Achenbach TM, & Rescorla L (2000). Manual for the ASEBA preschoool forms & profiles. University of Vermont, Research Centre for Children, Youth, & Families.

[R5] Achenbach TM, & Rescorla L (2001). Manual for the ASEBA school-age forms & profiles: An integrated system of multi-informant assessment. Aseba.

[R6] Allen JP, Manning N, & Meyer J (2010). Tightly linked systems: Reciprocal relations between maternal depressive symptoms and maternal reports of adolescent externalizing behavior. Journal of Abnormal Psychology, 119(4), 825–835. 10.1037/a0021081 21090880 PMC3057647

[R7] Anderson LS (2008). Predictors of parenting stress in a diverse sample of parents of early adolescents in high-risk communities. Nursing Research, 57(5), 340–350. 10.1097/01.NNR.0000313502.92227.87 18794718 PMC2706002

[R8] Anthony LG, Anthony BJ, Glanville DN, Naiman DQ, Waanders C, & Shaffer S (2005). The relationships between parenting stress, parenting behaviour and preschoolers’ social competence and behaviour problems in the classroom. Infant and Child Development, 14(2), 133–154. 10.1002/icd.385

[R9] Arikan G, Kumru A, Korkut B, & Ilhan AO (2019). Examining toddlers’ problem behaviors: The role of SES, parenting stress, perceived support and negative intentionality. Journal of Child and Family Studies, 28(12), 3467–3478. 10.1007/s10826-019-01529-y

[R10] Barroso NE, Mendez L, Graziano PA, & Bagner DM (2018). Parenting stress through the lens of different clinical groups: A systematic review & meta-analysis. Journal of Abnormal Child Psychology, 46(3), 449–461. 10.1007/s10802-017-0313-6 28555335 PMC5725271

[R11] Beernink ACE, Swinkels SH, Van der Gaag RJ, & Buitelaar JK (2012). Effects of attentional/hyperactive and oppositional/aggressive problem behaviour at 14 months and 21 months on parenting stress. Child and Adolescent Mental Health, 17(2), 113–120. 10.1111/j.1475-3588.2011.00616.x 32847294

[R12] Belsky J (1984). The determinants of parenting: A process model. Child Development, 55(1), 83–96. 10.2307/1129836 6705636

[R13] Berryhill MB, & Durtschi JA (2017). Understanding single mothers’ parenting stress trajectories. Marriage & Family Review, 53(3), 227–245. 10.1080/01494929.2016.1204406

[R14] Carapito E, Ribeiro MT, Pereira AI, & Roberto MS (2018). Parenting stress and preschoolers’ socio-emotional adjustment: The mediating role of parenting styles in parent-child dyads. Journal of Family Studies, 26(4), 594–610. 10.1080/13229400.2018.1442737

[R15] Cherry KE, Gerstein ED, & Ciciolla L (2019). Parenting stress and children’s behavior: Transactional models during Early Head Start. Journal of Family Psychology, 33(8), 916–926. 10.1037/fam0000574 31343210

[R16] Choi JK, & Becher EH (2019). Supportive coparenting, parenting stress, harsh parenting, and child behavior problems in nonmarital families. Family Process, 58(2), 404–417. 10.1111/famp.12373 29924390

[R17] Cicchetti D, & Schneider-Rosen K (1984). Toward a transactional model of childhood depression. New Directions for Child Development, 26, 5–27. 10.1002/cd.23219842604

[R18] Clark LA, & Watson D (2019). Constructing validity: New developments in creating objective measuring instruments. Psychological Assessment, 31(12), 1412–1427. 10.1037/pas0000626 30896212 PMC6754793

[R19] Conger KJ, Rueter MA, & Conger RD (2000). The role of economic pressure in the lives of parents and their adolescents: The family stress model. In Crockett LJ & Silbereisen RK (Eds.), Negotiating adolescence in times of social change (pp. 201–223). Cambridge University Press.

[R20] Crnic KA, Gaze C, & Hoffman C (2005). Cumulative parenting stress across the preschool period: Relations to maternal parenting and child behaviour at age 5. Infant and Child Development, 14(2), 117–132. 10.1002/icd.384

[R21] Damaske S, Bratter JL, & Frech A (2017). Single mother families and employment, race, and poverty in changing economic times. Social Science Research, 62, 120–133. 10.1016/j.ssresearch.2016.08.008 28126093 PMC5300078

[R22] Deater-Deckard K (1998). Parenting stress and child adjustment: Some old hypotheses and new questions. Clinical Psychology: Science and Practice, 5(3), 314–332. 10.1111/j.1468-2850.1998.tb00152.x

[R23] Deater-Deckard K, & Scarr S (1996). Parenting stress among dual-earner mothers and fathers: Are there gender differences? Journal of Family Psychology, 10(1), 45–49. 10.1037/0893-3200.10.1.45

[R24] Domitrovich CE, Durlak JA, Staley KC, & Weissberg RP (2017). Social-emotional competence: An essential factor for promoting positive adjustment and reducing risk in school children. Child Development, 88(2), 408–416. 10.1111/cdev.12739 28213889

[R25] Duncan R, Washburn IJ, Lewis KM, Bavarian N, DuBois DL, Acock AC, Vuchinich S, & Flay BR (2017). Can universal SEL programs benefit universally? Effects of the positive action program on multiple trajectories of social-emotional and misconduct behaviors. Prevention Science, 18(2), 214–224. 10.1007/s11121-016-0745-1 28028741 PMC5247357

[R26] Escudero V, & Friedlander ML (2017). Disadvantaged, multi-stressed families adrift in a sea of professional helpers. In Escudero V & Friedlander ML (Eds.), Therapeutic alliances with families (pp. 127–155). Springer. 10.1007/978-3-319-59369-2_6

[R27] Harmeyer E, Ispa JM, Palermo F, & Carlo G (2016). Predicting self-regulation and vocabulary and academic skills at kindergarten entry: The roles of maternal parenting stress and mother-child closeness. Early Childhood Research Quarterly, 37, 153–164. 10.1016/j.ecresq.2016.05.001

[R28] Hastings RP (2002). Parental stress and behaviour problems of children with developmental disability. Journal of Intellectual and Developmental Disability, 27(3), 149–160. 10.1080/1366825021000008657

[R29] Hill R (1958). Generic features of families under stress. Social Casework, 39(2–3), 139–150. 10.1177/1044389458039002-318

[R30] Hobbs S, & King C (2018). The unequal impact of food insecurity on cognitive and behavioral outcomes among 5-year-old urban children. Journal of Nutrition Education and Behavior, 50(7), 687–694. 10.1016/j.jneb.2018.04.003 29753634

[R31] Hofferth S, Davis-Kean PE, Davis J, & Finkelstein J (1999). 1997 user guide: The child development supplement to the panel study of income dynamics. Institute for Social Research, The University of Michigan.

[R32] Izquierdo-Sotorrío E, Holgado-Tello FP, & Carrasco MÁ (2016). Incremental validity and informant effect from a multi-method perspective: Assessing relations between parental acceptance and children’s behavioral problems. Frontiers in Psychology, 7, 664. 10.3389/fpsyg.2016.00664 27242582 PMC4861845

[R33] Jackson AP, & Choi JK (2018). Parenting stress, harsh parenting, and children’s behavior. Journal of Family Medicine & Community Health, 5(3), 1150–1159.

[R34] Jackson AP, Choi JK, & Preston KS (2019). Harsh parenting and Black boys’ behavior problems: Single mothers’ parenting stress and nonresident fathers’ involvement. Family Relations, 68(4), 436–449. 10.1111/fare.12373

[R35] Jackson AP, & Huang CC (2000). Parenting stress and behavior among single mothers of preschoolers: The mediating role of self-efficacy. Journal of Social Service Research, 26(4), 29–42. 10.1080/01488370009511335

[R36] Jocson RM, & McLoyd VC (2015). Neighborhood and housing disorder, parenting, and youth adjustment in low-income urban families. American Journal of Community Psychology, 55(3–4), 304–313. 10.1007/s10464-015-9710-6 25753403

[R37] Kochanova K, Pittman LD, & McNeela L (2022). Parenting stress and child externalizing and internalizing problems among low-income families: Exploring transactional associations. Child Psychiatry & Human Development, 53(1), 76–88. 10.1007/s10578-020-01115-0 33398689

[R38] Lee D, & Jackson M (2017). The simultaneous effects of socioeconomic disadvantage and child health on children’s cognitive development. Demography, 54(5), 1845–1871. 10.1007/s13524-017-0605-z 28836169 PMC5856460

[R39] Lee CYS, Lee J, & August GJ (2011). Financial stress, parental depressive symptoms, parenting practices, and children’s externalizing problem behaviors: Underlying processes. Family Relations, 60(4), 476–490. 10.1111/j.1741-3729.2011.00656.x

[R40] Little RJ (1988). A test of missing completely at random for multivariate data with missing values. Journal of the American Statistical Association, 83(404), 1198–1202.

[R41] Lunsky Y, Hastings RP, Weiss JA, Palucka AM, Hutton S, & White K (2017). Comparative effects of mindfulness and support and information group interventions for parents of adults with autism spectrum disorder and other developmental disabilities. Journal of Autism and Developmental Disorders, 47(6), 1769–1779. 10.1007/s10803-017-3099-z 28374207

[R42] Mackler JS, Kelleher RT, Shanahan L, Calkins SD, Keane SP, & O’Brien M (2015). Parenting stress, parental reactions, and externalizing behavior from ages 4 to 10. Journal of Marriage and Family, 77(2), 388–406. 10.1111/jomf.12163 26778852 PMC4712732

[R43] Morgan J, Robinson D, & Aldridge J (2002). Parenting stress and externalizing child behaviour. Child & Family Social Work, 7, 219–225. 10.1046/j.1365-2206.2002.00242.x

[R44] Muthén LK, & Muthén B (1998–2017). Mplus user’s guide (8th ed.). Muthén & Muthén.

[R45] Neece C, & Baker B (2008). Predicting maternal parenting stress in middle childhood: The roles of child intellectual status, behaviour problems and social skills. Journal of Intellectual Disability Research, 52(12), 1114–1128. 10.1111/j.1365-2788.2008.01071.x 18513339 PMC2787629

[R46] Neece CL, Green SA, & Baker BL (2012). Parenting stress and child behavior problems: A transactional relationship across time. American Journal on Intellectual and Developmental Disabilities, 117(1), 48–66. 10.1352/1944-7558-117.1.48 22264112 PMC4861150

[R47] Newman DA (2014). Missing data: Five practical guidelines. Organizational Research Methods, 17(4), 372–411. 10.1177/1094428114548590

[R48] Orth U, Clark DA, Donnellan MB, & Robins RW (2021). Testing prospective effects in longitudinal research: Comparing seven competing cross-lagged models. Journal of Personality and Social Psychology, 120(4), 1013–1034. 10.1037/pspp0000358 32730068 PMC7854859

[R49] Parent J, McKee LG, Rough JN, & Forehand R (2016). The association of parent mindfulness with parenting and youth psychopathology across three developmental stages. Journal of Abnormal Child Psychology, 44(1), 191–202. 10.1007/s10802-015-9978-x 25633828 PMC4520790

[R50] Parkes A, & Sweeting H (2018). Direct, indirect, and buffering effects of support for mothers on children’s socioemotional adjustment. Journal of Family Psychology, 32(7), 894–903. 10.1037/fam0000438 30091624 PMC6205417

[R51] Pelchat D, Bisson J, Bois C, & Saucier JF (2003). The effects of early relational antecedents and other factors on the parental sensitivity of mothers and fathers. Infant and Child Development, 12(1), 27–51. 10.1002/icd.335

[R52] Piedmont RL (2014). Inter-item correlations. In Michalos AC (Ed.), Encyclopedia of quality of life and well-being research. Springer. 10.1007/978-94-007-0753-5_1493

[R53] Rodriguez G, Hartley SL, & Bolt D (2019). Transactional relations between parenting stress and child autism symptoms and behavior problems. Journal of Autism and Developmental Disorders, 49(5), 1887–1898. 10.1007/s10803-018-3845-x 30623270 PMC6897296

[R54] Sameroff AJ, & Chandler MJ (1975). Reproductive risk and the continuum of caretaking casualty. In Horowitz FD, Hetherington M, Scarr–Salapatek S, & Siegal G (Eds.), Review of child development research (pp. 187–244). University of Chicago Press.

[R55] Sameroff AJ, & Fiese BH (2000). Transactional regulation: The developmental ecology of early intervention. In Shonkoff JP & Meisels SJ (Eds.), Handbook of early childhood intervention (pp. 135–159). University Press.

[R56] Sameroff AJ, & Mackenzie MJ (2003). Research strategies for capturing transactional models of development: The limits of the possible. Development and Psychopathology, 15(3), 613–640. 10.1017/S0954579403000312 14582934

[R57] Solem MB, Christophersen KA, & Martinussen M (2011). Predicting parenting stress: Children’s behavioural problems and parents’ coping. Infant and Child Development, 20(2), 162–180. 10.1002/icd.681

[R58] StataCorp. (2015). Stata statistical software: Release 14 [computer software]. StataCorp LLC.

[R59] Stone LL, Mares SH, Otten R, Engels RC, & Janssens JM (2016). The co-development of parenting stress and childhood internalizing and externalizing problems. Journal of Psychopathology and Behavioral Assessment, 38(1), 76–86. 10.1007/s10862-015-9500-3 27069304 PMC4789299

[R60] Taris TW, & Kompier MAJ (2014). Cause and effect: Optimizing the designs of longitudinal studies in occupational health psychology. Work & Stress, 28(1), 1–8. 10.1080/02678373.2014.878494

[R61] Tervo RC (2012). Developmental and behavior problems predict parenting stress in young children with global delay. Journal of Child Neurology, 27(3), 291–296. 10.1177/0883073811418230 21968980

[R62] The Trustees of Princeton University (2021). About the fragile families and child wellbeing study. The Trustees of Princeton University. https://fragilefamilies.princeton.edu/about

[R63] Turner KM, & Sanders MR (2006). Dissemination of evidence-based parenting and family support strategies: Learning from the Triple P—Positive Parenting Program system approach. Aggression and Violent Behavior, 11(2), 176–193. 10.1016/j.avb.2005.07.005

[R64] Wang D, Choi JK, & Shin J (2020a). Long-term neighborhood effects on adolescent outcomes: mediated through adverse childhood experiences and parenting stress. Journal of Youth and Adolescence, 49(10), 2160–2173. 10.1007/s10964-020-01305-y 32804295

[R65] Wang X, Maguire-Jack K, Barnhart S, Yoon S, & Li Q (2020b). Racial differences in the relationship between neighborhood disorder, adverse childhood experiences, and child behavioral health. Journal of Abnormal Child Psychology, 48(3), 315–329. 10.1007/s10802-019-00597-4 31811546

[R66] Ward KP, & Lee SJ (2020). Mothers’ and fathers’ parenting stress, responsiveness, and child wellbeing among low-income families. Children and Youth Services Review, 116, 105–218. 10.1016/j.childyouth.2020.105218 PMC7425837 32801410

[R67] Williford AP, Calkins SD, & Keane SP (2007). Predicting change in parenting stress across early childhood: Child and maternal factors. Journal of Abnormal Child Psychology, 35(2), 251–263. 10.1007/s10802-006-9082-3 17186365

[R68] Woodman AC (2014). Trajectories of stress among parents of children with disabilities: A dyadic analysis. Family Relations, 63(1), 39–54. 10.1111/fare.12049

[R69] Woodman AC, Mawdsley HP, & Hauser-Cram P (2015). Parenting stress and child behavior problems within families of children with developmental disabilities: Transactional relations across 15 years. Research in Developmental Disabilities, 36, 264–276. 10.1016/j.ridd.2014.10.011 PMC4425632 25462487

[R70] Yan N, Ansari A, & Peng P (2021). Reconsidering the relation between parental functioning and child externalizing behaviors: A meta-analysis on child-driven effects. Journal of Family Psychology, 35(2), 225–235. 10.1037/fam0000805 33104378

[R71] Zisser A, & Eyberg SM (2010). Parent-child interaction therapy and the treatment of disruptive behavior disorders. In Weisz JR & Kazdin AE (Eds.), Evidence-based psychotherapies for children and adolescents (pp. 179–193). The Guilford Press.

